# The gaze of a social monkey is perceptible to conspecifics and predators but not prey

**DOI:** 10.1098/rspb.2022.0194

**Published:** 2022-06-08

**Authors:** Will Whitham, Steven J. Schapiro, Jolyon Troscianko, Jessica L. Yorzinski

**Affiliations:** ^1^ Department of Ecology and Conservation Biology, Texas A&M University, College Station, TX, USA; ^2^ Department of Comparative Medicine, UT MD Anderson Cancer Center, Bastrop, TX, USA; ^3^ Centre for Ecology and Conservation, University of Exeter, Exeter, UK

**Keywords:** colour vision, eye morphology, gaze perception, iris, sclera, tufted capuchin

## Abstract

Eye gaze is an important source of information for animals, implicated in communication, cooperation, hunting and antipredator behaviour. Gaze perception and its cognitive underpinnings are much studied in primates, but the specific features that are used to estimate gaze can be difficult to isolate behaviourally. We photographed 13 laboratory-housed tufted capuchin monkeys (*Sapajus [Cebus] apella*) to quantify chromatic and achromatic contrasts between their iris, pupil, sclera and skin. We used colour vision models to quantify the degree to which capuchin eye gaze is discriminable to capuchins, their predators and their prey. We found that capuchins, regardless of their colour vision phenotype, as well as their predators, were capable of effectively discriminating capuchin gaze across ecologically relevant distances. Their prey, in contrast, were not capable of discriminating capuchin gaze, even under relatively ideal conditions. These results suggest that specific features of primate eyes can influence gaze perception, both within and across species.

## 1. Introduction

Discrimination of animal gaze probably plays many pivotal roles in animal ecology. Within a species, the gaze of conspecifics can be very informative. Gaze can cue an individual to the presence of a threat, as when animals as disparate as primates [1], goats (*Capra hircus*) [2] and red-footed tortoises (*Geochelone carbonaria*) [3] follow the gaze of a conspecific that looks up (i.e. to a flying predator) [4]. Gaze can also be used to maximize available food in competitive foraging or caching, as when chimpanzees (*Pan troglodytes*) [5], common marmosets (*Callithrix jacchus*) [6] and scrub-jays (*Aphelocoma californica*) [7] cache or exploit food resources at times when competitors cannot see them. Gaze can perform a social function as well, as when primates use directed gaze as a threat that only an averted gaze in reply can serve to assuage [8].

Across species, discrimination of the direction in which another species's eyes, head or body are oriented can inform decision-making of predators and prey alike [9,10]. Because predators risk energy, injury and even death when exploiting certain prey, they probably use many information sources to estimate the risk associated with a predation attempt. Eye gaze appears to be one such source of information, as when white-tailed deer (*Odocoileus virginanus*) stare at coyotes (*Canis latrans*) [11]. Exaggerated, concentric circles that resemble eyes on the wings of butterflies and the fins of tropical fishes are more successful than other patterns at preventing predation also suggests that predators attend to eyes [12,13]. Conversely, prey can attend to predator gaze to estimate the likelihood of an imminent predation attempt. If the predator is not oriented in the direction of the prey, the energy expenditure associated with an attempt to flee may be unnecessarily costly. This principle, too, is well validated by behavioural observations, as when Indian rock lizards (*Psammophilus dorsalis*) [14], magpies (*Pica pica*) [15] and European starlings (*Sturnus vulgaris*) [16] monitor the gaze direction of human threats prior to making the decision to flee. Taken together, a species that is both predator to and prey of other species would optimally exhibit a gaze that is simultaneously discriminable to its predators (to dissuade possible predation events) and inconspicuous to its prey (to camouflage intent when hunting).

Primates are a good model for the study of the eyes' contribution to animal gaze perception: primate faces exhibit a remarkable diversity of colours (and eye colours are particularly variable), they are phenotypically diverse in how they perceive colour, and their ecology suggests potentially important roles of gaze signalling, both within and across species [17–20]. In primates for which gaze serves an agonistic function, directed/averted gaze can be used as an additional set of cues for prediction of whether appetitive or reproductive resources can be safely exploited, or whether a subordinate animal's insurrection against a dominant animal would be supported. Whether primates use the direction of gaze in service of more cognitively complex social behaviours is less clear. Yamagiwa [21] analysed staring behaviours of a bachelor group of mountain gorillas (*Gorilla beringei beringei*), and reported that these episodes functioned to initiate play, sexual activity and post-conflict reconciliation. Like other mammals, primates demonstrate stimulus enhancement effects, such that a conspecific's directed behaviours toward a stimulus causes observers to be more likely to interact with that stimulus than non-observers [22,23]. Primates cooperate to solve shared problems, including playing an active role in recruiting prospective cooperative partners, and both cooperate and recruit more effectively when they can see the conspecific with which they are cooperating [24,25].

The extent to which primates use gaze can be difficult to assess behaviourally. Many species of prosimians, monkeys and apes can follow eye gaze (for a review see [26]). Unfortunately, these designs often use human experimenter eye gaze as the gaze to-be-followed. Since animals are exceptionally skilled at learning the subtle cues of human experimenters to predict reinforcement (e.g. Clever Hans effects), gaze following may be interpreted at least equally effectively as a test of the animal's general ability to act on cues (including but not limited to eye gaze) from their experimenters [27]. Designs in which the cues to be followed are not those of a human experimenter may provide better evidence of nonhuman primate attention to gaze. Tomasello *et al.* [1] demonstrated that each of five primate species from disparate genera would orient to a remote experimenter after being cued to do so by a confederate conspecific. This demonstration, in which each species followed conspecific gaze at high rates in a relatively natural setting (the animals moved and behaved freely as part of large social groups), suggests that gaze following may indeed be a behaviour well-distributed across primates. Computerized experiments that used digital photos or videos of conspecifics to cue gaze following behaviours suggest the same [28,29].

To clarify the specific cues involved with any gaze perception, Tomasello *et al.* [30] independently varied experimenter eye direction and head direction and found that head direction was much more predictive of chimpanzee looking behaviour than was eye direction, whereas the opposite was true of human infants. In a design that used digital images of rhesus macaques with averted eyes or heads as predictive cues for eye saccades to a target stimulus, Deaner & Platt [28] reported that averted eyes were as effective a cue as an averted head for this species (although targets to the cued side were detected only approximately 5 ms faster than targets to the uncued side). Recently, Kano *et al*. [31] demonstrated that captive chimpanzees (*Pan troglodytes*) can discriminate between directed and averted eyes [31]. In sum, nonhuman primates use the information of conspecific gaze, but the cues that they use to make these judgements are little understood and not easily investigated in laboratory settings.

In order to estimate gaze direction a receiver must be able to compare the locations of the pupil and sclera. This task, therefore, depends on an interaction between receiver vision and the colours, sizes and shapes of the regions of the eye [32–34]. Human eyes, with white sclera, iris and pupil that contrast with the sclera, and eyes that appear wider than they are tall, are uniquely well-suited to reveal gaze direction to conspecifics [20,35]. Accordingly, detection and utilization of human conspecific gaze is of critical importance for shared attention [36]. By contrast, the gaze direction of other primate species may be obscured to various degrees by the shape of the eye, by the low degree of contrast between the species' sclera and iris colour, and by the species' sensitivity to colours (e.g. dichromatic versus trichromatic) [20].

Determining whether gaze cues are visible to a given receiver, therefore, requires visual modelling that takes into account both contrast/coloration and spatial vision (the distance of the receiver and size of the visual cue). Recent advances in spatiochromatic visual modelling techniques allow us to perform this for nonhuman receivers [37–40]. An objective analysis of the degree to which different species have the capacity to discriminate and potentially use gaze information requires both an objective measurement of the colours involved in gaze assessment and an understanding of how the receiver species interprets colours. Previous work has not accounted for differences in vision across species nor used colour-calibrated photographs [41–43].

Our study quantifies the eye coloration of a New World monkey species, tufted capuchin monkeys (*Cebus [Sapajus] apella*; hereafter capuchins), as they would appear to conspecifics, their prey, and their predators at a range of simulated distances. Capuchins' ecological niche as a group-living primate that is both predator and prey makes them an ideal model species for our study. In the wild, these animals live in large social groups and evidence complex social behaviours, like cooperative hunting and tool use, for which gaze perception likely conveys important information [44]. Capuchins both flee from predators and cooperate with conspecifics to confront them, and conspecific eye gaze may serve as an important cue for estimating predator location in service of coordinating these behaviours [45]. Socially dominant capuchins may prevent subordinate animals from exploiting food resources, and conspecific eye gaze may serve as a cue to whether food can be approached safely (as when common marmosets surreptitiously exploit food that a dominant animal cannot see) [6]. Finally, capuchins (like most New World monkeys) are unusual in that they can either be dichromatic or trichromatic (hereafter identified by the wavelengths at which their two or three colour receptors are most sensitive, e.g. 426–536, 426–536–561, etc.) [46]. We thus hypothesize that, across vision phenotypes, capuchins will be able to discriminate conspecific gaze for use in these varied social contexts and others.

Capuchins also prey on other animals, including coatimundis (*Nasua narica*) and small birds [44,47]. We hypothesize that the gaze of capuchins should appear camouflaged to their prey so that the prey cannot determine whether the capuchins have detected them. A camouflaged gaze can act as an important determinant of prey behaviour. For birds, lizards and small mammals, it is the direction in which a capuchin gazes that drives decisions to flee, rather than mere proximity to the prospective predator [14–16]. For this reason, capuchins are probably more successful in hunting when their prey are unaware that they have been detected [48]. Finally, capuchins are predated by harpy eagles (*Harpia harpyja*), with capuchins making up 18% of harpy eagle diets [49–51]. We hypothesize that the eyes of capuchins (alongside other cues like head or body orientation) should appear conspicuous to their predators so that their predators can determine that the capuchins have detected them. Capuchins are more likely to avoid being attacked by signalling to predators that the predators have been detected [48].

To test our hypotheses, we measured 131 iris, 109 pupil, 79 sclera and 131 skin regions of interest (ROI) from 131 photographs of 13 tufted capuchin monkeys (1–29 photographs per animal), then estimated how discriminable these regions would be to each of nine visual systems (three capuchin dichromat, three capuchin trichromat, coatimundi, small bird and eagle) at each of nine simulated distances ranging from 0.25 to 64 m. In doing so, we provide an analytical demonstration of eye colour's contribution to a species' ecology.

## 2. Results

Animals can make discriminations on the basis of chromatic information, achromatic information or both. We used ΔS values to quantify how discriminable one ROI is from another for any given vision phenotype and distance. A ΔS value greater than some criterion (here, 3 ΔS) predicts that two ROIs are likely to be discriminable, whereas a ΔS value less than the criterion suggests that the two ROIs are not likely to be discriminable. We computed chromatic and achromatic ΔS estimates for up to six contrasts among the ROIs (i.e. iris–pupil, iris–sclera, etc.), at nine simulated distances, for nine visual systems, for each of 131 photographs of capuchins, and estimated uncertainty around these estimates using one Bayesian regression for chromatic data and another for achromatic data.

**Figure 1.**
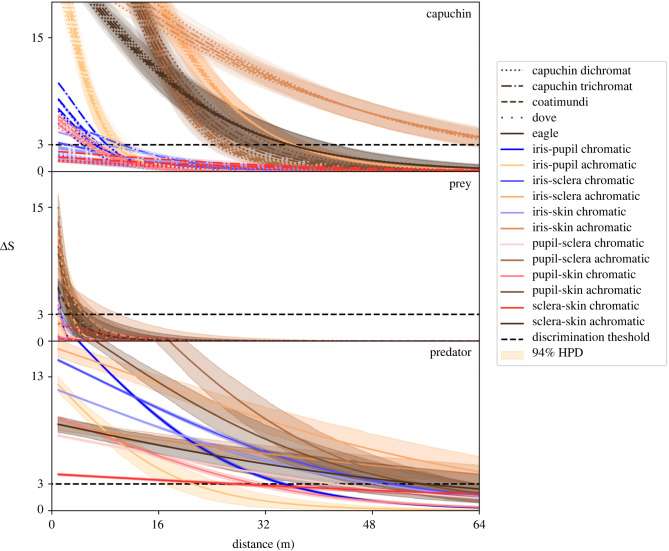
Regression estimates for chromatic and achromatic ΔS as a function of simulated distance, colour vision phenotype and ROI contrast. Chromatic contrasts are in shades of blue and red, achromatic contrasts are in shades of brown, and visual systems are identified by different line styles (one line each for the three dichromat and three trichromat capuchin vision phenotypes). (Online version in colour.)

Chromatic and achromatic ΔS discriminability values without acuity correction suggest that the chromatic and achromatic contrasts of capuchin gaze are often discriminable (electronic supplementary material, text and figures S2–S4; distance = 0 on figure 1). The more relevant measure is how discriminable gaze is at distances that are relevant to capuchin ecology. Both chromatic and achromatic ΔS values decreased as simulated distance increased, and this effect was strongest for contrasts involving the pupil (the first ROI contrasts to become indiscriminable).

Both chromatic and achromatic estimates suggest that capuchins, regardless of their vision phenotype, can discriminate gaze at many simulated distances (top panel of figure 1). A Bayesian 94% highest posterior density interval (HPD) around ΔS that contained 94% of the most probable values for ΔS did not include the discrimination threshold at short simulated distances for any achromatic ROI contrast of every capuchin vision phenotype, suggesting (at *α* = 0.06) that monkeys can discriminate these ROI contrasts. Chromatic HPD exceeded the discrimination threshold at short simulated distances for all capuchin vision phenotypes when the pupil was involved in a discrimination, and for trichromatic phenotypes on the additional ROI contrast between iris and sclera. Similarly, eagles can probably discriminate capuchin gaze at many simulated distances (bottom panel of figure 1): chromatic and achromatic HPD that do not include the discrimination threshold until extreme distances suggest a high degree of ability to discriminate capuchin gaze. This is especially true for ROI contrasts among iris, sclera and skin. By contrast to the capuchins and eagles, it is unlikely that prey (small birds and coatimundi) are able to reliably discriminate capuchin gaze, with chromatic and achromatic HPD that uniformly include the discrimination threshold beyond the shortest simulated distances (middle panel of figure 1).

**Figure 2.**
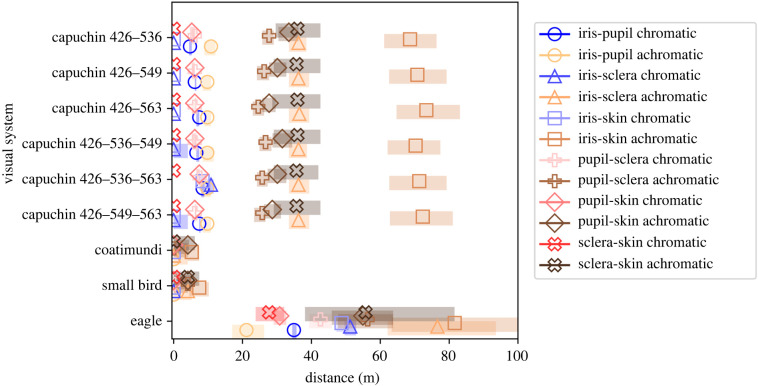
Estimates of the distance at which an ROI contrast would no longer be discriminable for each phenotype on chromatic and achromatic bases. Shading denotes the bounds of the 94% HPD around each estimate. (Online version in colour.)

Estimates of the distance at which ΔS values fall below the discrimination threshold are presented in figure 2. Gaze discrimination by capuchins and eagles may be possible at up to several dozen meters, primarily on an achromatic basis (figure 2). For all comparisons, achromatic ΔS values were greater than chromatic ΔS values, due to the relative lack of chromatic signal in the blacks, whites and browns of capuchin coloration.

## 3. Discussion

We found support for our hypotheses that the gaze of a social primate species is discriminable to conspecifics and their predators but not their prey. Based on chromatic and achromatic contrasts between the iris, pupil, sclera and skin, it is likely that capuchins can reliably discriminate the eye gaze of other capuchins regardless of their specific colour vision phenotype. This is probably the case at both short and long distances. Similarly, a major predator of capuchins (harpy eagles) can also probably discriminate the eye gaze of capuchins even at long distances. By contrast, prey species (coatimundi and small birds) are unlikely to be able to discriminate capuchin eye gaze at even very short distances. The pattern of capuchin gaze discriminability that our analyses suggest is uniquely beneficial to the capuchins: the capuchins have access to the gaze information of their conspecifics, they can signal to their raptor predators that the predators have been detected, and they do not readily reveal their gaze to prey. These results are, to our knowledge, the first application of validated techniques to examine primate eye colour.

Capuchins can discriminate eye gaze similarly regardless of their specific vision phenotype. This is because chromatic information of the capuchin gaze was much less variable than the achromatic information, and trichromatic and dichromatic monkeys discriminate achromatic information of the capuchin eyes nearly identically. Trichromatic animals' advantages in gaze discrimination were subtle, and largely limited to chromatic contrasts involving the iris. Therefore, the colour vision phenotypes of capuchins likely evolved for other contexts, such as foraging [52].

Gaze perception is also likely important during interspecific interactions. Even though vertebrate prey are not the primary food source for capuchins (less than 10%) [53], capuchins probably have higher success when hunting if prey cannot detect their eye gaze [48]. Similarly, capuchins can potentially dissuade predatory attacks by signalling to their predators that the predators have been detected [48]. We would predict that capuchins with eyes directed toward a predator are less likely to experience a predation attempt than are capuchins without their eyes directed to the predator. Because prey could not discriminate capuchin gaze, we would predict that they, like other prey species, use more discriminable cues (e.g. head direction, as in some birds [16]), other senses (e.g. olfaction [54]) or both when evaluating whether to flee from predators.

Colour vision modelling is best applied to comparisons among similarly sized regions, in daylight and against a neutral background colour [55]. These ideal conditions are unlikely in many actual signalling contexts (i.e. rainforest animals looking at shaded capuchin monkeys against a potentially variegated background). Our analyses thus estimate species' ability to discriminate capuchin gaze under relatively ideal conditions; in the wild, their discriminations of capuchin gaze may be more limited than our analyses suggest. Even in this case of ideal viewing circumstances, the modelling suggested that prey species could not effectively discriminate capuchin gaze. Other contexts in which gaze discrimination may be important adhere closely to these ideal conditions. Because capuchin nut cracking appears to involve relatively bright, diffuse lighting, it is possible that the monkeys' ability to discriminate conspecific gaze is best (i.e. most like our models) at this time, in which the ability to socially learn from conspecifics is at a premium [56]. Similarly, the same conditions that are likely to facilitate an eagle identifying a capuchin—the capuchin emerging from forest cover in daylight—would also facilitate the eagle's discrimination of whether or not it has been detected by the capuchin.

Assumptions made within our models may limit the conclusions of our results. In the colour vision models, achromatic RNL models are not yet well supported by accompanying behavioural data on animal achromatic discrimination thresholds [40]. It is possible that with either additional behavioural support or new methods for discerning luminance discrimination, our methods will be found to over- or under-estimate our species's capacity to discriminate eyes achromatically. Despite these limitations, our methods are the standard of colour ecology research. They are singular in their attention to control over potential biases in digital images, how colour would be perceived by diverse species under study, and the ecological context in which the visual information would be viewed. This analytical approach makes at least two contributions. First, our quantification of the colours of capuchin gaze is an initial datapoint in describing the variability of primate eye colours that can function in both intraspecific and interspecific contexts [20]. For primates living in large social groups, gaze may be more discriminable and more likely to serve a communicative function during intraspecific interactions compared to primate species living in smaller groups. For other primates, gaze may be camouflaged, potentially to conceal information. Second, our estimation of the discriminability of these colours as they would appear to different receivers is new to animal gaze perception research, and readily applicable to any species for which the relevant parameters can be estimated.

We estimated the discriminability of one species's gaze as it could apply to within- and between-species functions, but there are many other comparisons to be made. The relationships among predator and prey discrimination of gaze cues that our analyses reveal might be qualified or extended by future research with other genera. Complementary, behavioural experiments that quantify the degree to which different species use gaze information promise to better our understanding of gaze perception. Taken together, these future directions afford scientific breadth and depth to the study of animal eye colour and its underexplored contributions to the perceptual world of nonhuman animals.

## 4. Material and methods

### (a) Acquiring photos

The capuchins photographed for this study were socially housed in group sizes ranging from 4–8 conspecifics. The animals were not restricted calorically and ate a complete diet of fruit, vegetables and commercial primate chow. One researcher (W.W.) took photographs of the animals as they rested, foraged or exhibited other species-typical behaviours in large outdoor play yards. The monkeys were habituated to humans (including the researcher) and cameras, and they did not exhibit any unusual distress during photography.

We took precautions to minimize sources of image colour variability. We took the photographs as uncompressed RAW files (*.ARW) using a Sony *α*7ii [ILCE-m2] mirrorless digital camera with a Sony 30–70 mm lens. We used the same camera settings in all photographs: an aperture size of F/5.6, a focal length of 70 mm and an ISO of 3200. We took all photographs on clear days between 12.45 and 15.45 in the summer of 2020 at a distance of 1–3 m from the monkeys. Because camera shutter speed does not affect colour in photographs, we adjusted shutter speeds as needed to ensure proper exposure.

We calibrated the colour information in each image using a 20% reflectance grey standard (Spectralon; Labsphere, Inc.). Because inclusion of the grey standard in each photograph was not feasible when photographing unrestrained monkeys, we took a photograph of the grey standard in the same light conditions, and using the same camera settings, within minutes of each photograph of a monkey (i.e. the sequential method) [57,58].

### (b) Conversion to target species vision

#### (i) Initial processing

We calibrated and processed the photographs using the micaToolbox plugin (www.empiricalimaging.com) [39] for ImageJ (NIH, Bethesda, ML). Table 1 lists all species-specific calibration parameters. We used micaToolbox to import RAW, linear photographs, then normalize them via comparison with RAW photographs of the grey reflectance standard so that differences in light levels across photographs were controlled. Photographs were then converted into multispectral images to allow spectral sensitivity functions to be customized for each receptor type of the camera and each species of our study.

**Table 1.** Species-specific image calibration and modelling parameters.

species	wavelength of peak sensitivity for cone type			acuity (cyc/deg)	receptor noise		
	SW	MW	LW		SW	MW	LW
tufted capuchin monkey	426	—	536	46.8	0.08	—	0.02
	426	—	549	46.8	0.08	—	0.02
	426	—	563	46.8	0.08	—	0.02
	426	536	549	46.8	0.08	0.02	0.02
	426	536	563	46.8	0.08	0.02	0.02
	426	549	563	46.8	0.08	0.02	0.02
harpy eagle	477	537	605	140	∼0.054^a^	0.05	∼0.051^b^
small bird	453	539	607	7.6	∼0.059^c^	−0.05	0.05
coatimundi	433	—	554	5	0.22	—	0.05

^a^0.0538027587

^b^0.0511766316

^c^0.0592927061

#### (ii) Converting to cone catch using spectral sensitivities

We transformed the multispectral images from a state in which pixel values represented the spectral sensitivities of the camera to a state in which the pixel values represented the quantal catch of target species' cone receptors (i.e. a cone catch image). To make this transformation, we used both the spectral sensitivities of the camera and the spectral sensitivities of all target species. Spectral sensitivity information for the camera sensor was already included in the standard distribution of micaToolbox [39]. For animal spectral sensitivities, we used the opsin templates of Govardovskii *et al.* [59] to estimate the spectral sensitivities of each receptor type and each vision phenotype using only an estimate of the wavelength of light at which a receptor is most sensitive (for details on our estimation of spectral sensitivities, see electronic supplementary material, information SI). To transform images from camera colour space to animal colour space, cone mapping models were created using micaToolbox's ‘generate cone mapping model from spectral sensitivities' function and implemented using its ‘convert to cone catch’ function.

### (c) Simulated distances

**Figure 3.**
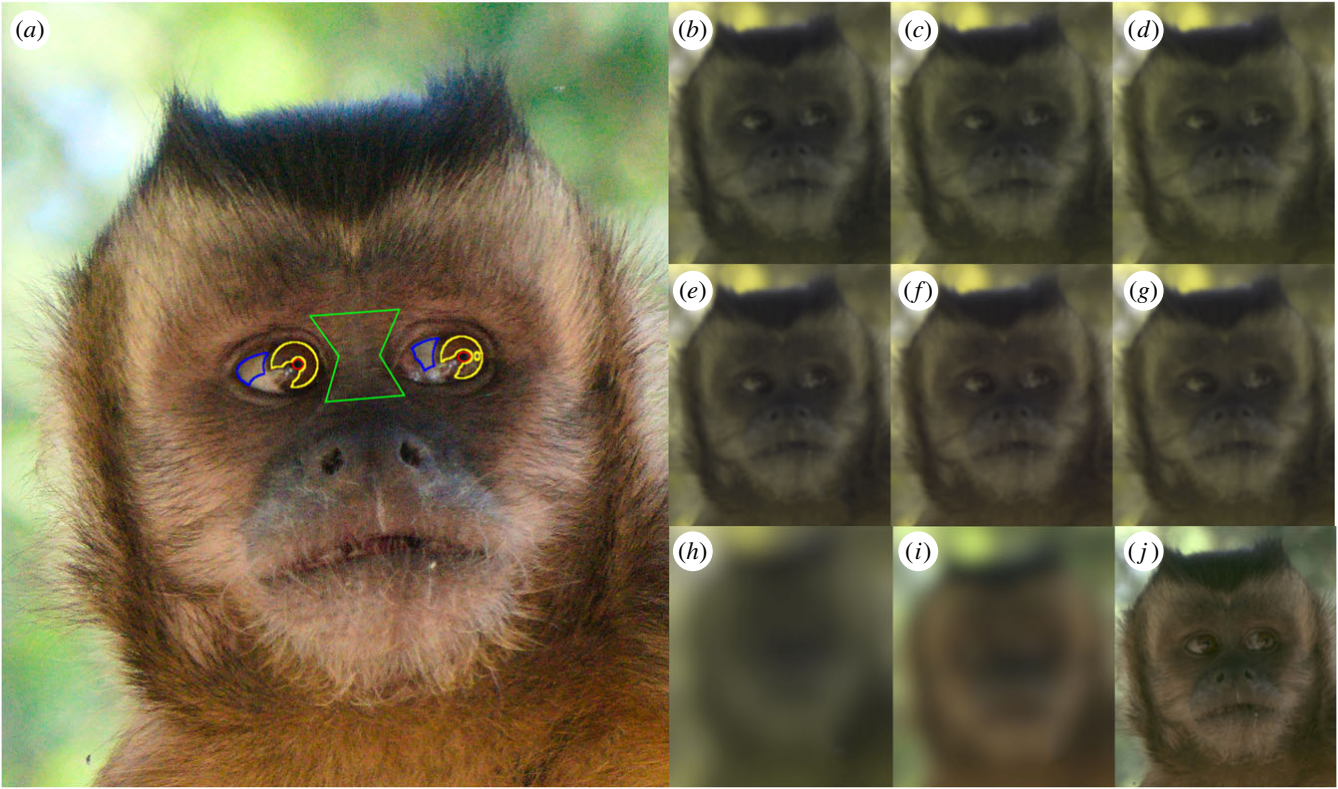
(*a*) Example regions of interest (ROIs). The red ROIs delineate the capuchin's pupils, the yellow ROIs delineate the irises, the blue ROIs delineate the sclera, the green ROI delineates the skin. Reflections in the ocular media were excluded from the ROIs. (*b*–*j*): A visualization of the colour and acuity corrections performed on photographs of capuchin faces at a simulated distance of 4 m. (*b*) capuchin 426–536, (*c*) capuchin 426–549, (*d*) capuchin 426–563, (*e*) capuchin 426–536–549, (*f*) capuchin 426-536-563, (*g*) capuchin 426–549–563, (*h*) coatimundi, (*i*) small bird and (*j*) eagle colour vision phenotypes. (Online version in colour.)

In order to simulate colour information as it would appear to the target species at different distances, images were degraded in accordance with the visual acuity of the target species using micaToolbox's version of AcuityView [37,39]. We used 15.1 mm as an estimate of the width of the capuchin eye for the scale bar for acuity corrections [17]. For details on our acuity correction, see electronic supplementary material, information SI. A visual representation of the combined effects of cone catch conversion and acuity corrections for each of the nine vision phenotypes is presented in figure 3.

### (d) Measurement and analysis of gaze

#### (i) Selection of regions of interest

A receiver's accurate discrimination of eye gaze direction likely depends on visual information from the signaller's iris, pupil, sclera and skin [20,32]. We created regions of interest (ROIs) by outlining these areas using ImageJ's selection tools. Not all ROIs were present in each photograph; in some photographs the sclera was not visible or the pupil was completely obscured by reflections in the ocular media. Primate ocular media can be very reflective, and we excluded highlights and reflections from ROIs. The skin ROI for each photo was created by using a polygon in the shape of an hourglass between the eyes of the monkeys (figure 3).

#### (ii) Receptor noise limited modelling

To determine whether each ROI is discriminable from another ROI for each vision phenotype, we used the receptor noise limited (RNL) model [55]. The RNL model assumes that for a light-adapted eye, the probability of successful cone receptor-based discriminations between similarly sized colours against a white background are a function of the visual information available, the noise in individual colour receptors, and the relative numbers of different types of colour receptors. RNL does not account for discriminations based on lines, textures, movement or contours. We used the micaToolbox macro ‘cone catch to RNL chromaticity’ to transform cone catch images to RNL colour space and measure ROIs [39]. This RNL model (hereafter: chromatic RNL) estimates performance in colour-based discriminations. For our analysis, quantification of any achromatic signalling is also important because much of the informational content of the ROI is not colour, with capuchin pupils, sclera and skin having relatively little chromatic information. For this reason, we estimated performance in luminance-based discriminations by additionally using an achromatic RNL model [60]. Species-specific modelling parameters for chromatic and achromatic RNL modelling are included in table 1. For details about and parameters for achromatic RNL modelling, see electronic supplementary material, information SI text.

The output of RNL modelling is a ΔS value that estimates how discriminable one ROI is from another (similar to just-noticeable-difference values) [61]. Because the just-noticeable-difference values of psychophysics research typically have extensive behavioural validations, whereas ΔS does not, we use the latter term exclusively. A ΔS value greater than some criterion (often 1 ΔS) predicts that two ROIs are likely to be discriminable, whereas a ΔS value less than the criterion suggests that the two ROIs are not likely to be discriminable. Valid estimates for ΔS can be any value greater than 0, but the predictive validity of ΔS as a measure of perceptual discriminability is largely limited to the comparison of the estimate with some criterion value. For example, from both suprathreshold ΔS values of 11 and 17, one would conclude that a comparison is discriminable since both values are greater than any typical discrimination threshold. We chose to use a conservative criterion of 3 ΔS as our discrimination threshold for all ROI contrasts.

#### (iii) Statistics

RNL modelling returned chromatic and achromatic ΔS estimates for up to six contrasts among the ROIs, at nine simulated distances, for nine colour vision phenotypes, for each of 131 photographs of capuchins. These estimates are based on mathematical modelling of the physiology and psychology of the target species and are appropriate for inference [61]. However, to extend these inferences beyond the simulated distances of all applied acuity corrections, and to quantify uncertainty around each ΔS in a way appropriate for comparison with our discriminability threshold, two Bayesian regressions were fitted: one for chromatic data and one for achromatic data. More specifically, we modelled each ΔS dataset as a logistic function of simulated distance. For more information on regression priors and procedures, and model diagnostics, see electronic supplementary material, information SI text.

## Data Availability

All data are available in an OSF repository at https://osf.io/6ad2n/. The data are provided in electronic supplementary material [62].
